# Guided endodontic treatment in a region of limited mouth opening: a case report of mandibular molar mesial root canals with dystrophic calcification

**DOI:** 10.1186/s12903-022-02067-8

**Published:** 2022-02-11

**Authors:** Marcos Coelho Santiago, Michel Mattar Altoe, Caroline Piske de Azevedo Mohamed, Laudimar Alves de Oliveira, Loise Pedrosa Salles

**Affiliations:** 1 Post-Graduation Program in Dentistry, Faculty of Health Sciences, University of Brasilia (UnB), Campus Universitário Darcy Ribeiro, Asa Norte, Brasília, DF 70910-900 Brazil; 2 Department of Dentistry, Faculty of Health Sciences, University of Brasilia (UnB), Campus Universitário Darcy Ribeiro, Asa Norte, Brasília, DF 70910-900 Brazil

**Keywords:** Guided endodontics, Template, Dystrophic calcification, Root canal treatment

## Abstract

**Background:**

The endodontic treatment of calcified root canals in molars is a challenging and time-consuming procedure. Even with the aid of a surgical microscope, the risk of root perforation is high, especially in the furcation area. The purpose of this study is to report the Computer-Aided-Design and Manufacturing (CAD–CAM) workflow, the innovative strategies for the template ideation, and the guided endodontic treatment of a mandibular molar with dystrophic calcification in the mesial root canals.

**Case presentation:**

A 58-year-old female patient, ASA I, was referred to endodontic treatment in the right first mandibular molar for prosthetic reasons. The mesiobuccal and mesiolingual canals appeared obliterated in the radiographic images. The absence of dental crown, tooth inclination, and the limited mouth opening of the region contributed to a poor visual reference of the tooth in the dental arch and the direction of the remaining lumens of the canals. Despite using surgical microscopy, the conventional technique led to the deviation of the mesiobuccal canal towards the furcation area. The obliteration of both mesial root canals was confirmed using the Cone Beam Computer Tomography. The clinical history associated with the tomography diagnosis was compatible with dystrophic calcifications in the pulp canals. The patient was submitted to an intra-oral scanning as well. The Digital Imaging and Communications in Medicine data (DICOM) were segmented. The Standard Tessellation Language (STL) files were processed following the CAD–CAM workflow, aiming to create two different endodontic templates with a new open design concept. The templates with open design allowed direct visualization of the operative field, irrigation, and dentin debris removal. The strategy of the guidance sleeves niche as half-cylinders allowed the drill insertion in a limited mouth opening region.

**Conclusions:**

The digital planning and guided access permitted to overcome the case limitations and then re-establish the glide path following the original anatomy of the root canals. The guided endodontic represents a personalized technique that provides security, reduced risks of root perforation, and a significant decrease of the working time to access obliterated root canals even in the mesial root canal of mandibular molars, a region of limited mouth opening.

## Background

The guided endodontics is a novel technique based on Computer-Aided-Design and Computer-Aided-Manufacturing (CAD–CAM) workflow to plan and create 3D-printed templates, which aim to guide the access of pulp canals with pathologic anatomy or obliterations. The first report of guided endodontics, that most approaches to the guided endodontic as currently known, referred to treating an upper central incisor with Dens Invaginatus [1]. Unlike current templates, the study's prototype was a 3D-printed jig fixed on the tooth with a channel to guide the penetration of the drill and the working length [1]. According to the authors, adopting an endodontic template promoted safety with a significant reduction in working time. Since then, guided endodontics is evolving and expanding its indications, especially for cases with pulp canal obliteration (PCO).

PCO demands innovative strategies to achieve treatment success in complex endodontic cases and seems to be inspiring a spread of the guided endodontics usage. Dental trauma as concussion and subluxation are considered the leading causes of PCO, also named calcific metamorphosis [2, 3]. Pulp canal obliteration is also found in individuals with genetic disorders of the connective tissue, such as the Syndrome of Ehlers-Danlos Type I [4]; type II dentin dysplasia [5]; tumoral calcinosis, Marfan's syndrome [6]; Osteogenesis imperfecta Type I and Otodental Syndrome [7]. Prolonged stimuli as caries, attrition, abrasion, erosion, surgical procedures, and orthodontic therapy can similarly lead to the obliteration of root canals by dystrophic calcifications and pulp stones [8]. Dystrophic calcification in the coronal region and root canal is prevalent in older individuals, which might be due to the pulp aging process and the reduced pulpal blood flow [9]. The deposition of secondary and tertiary dentin during the aging process causes the constriction of the pulp chamber space and, consequently, difficulties when endodontic treatment is in need. Negotiating a narrow, curved, and calcified root canal often results in an increased risk of perforation of the floor of the pulp chamber, lateral wall, and root [10, 11]. The cone-beam computed tomography proved to be an efficient method for diagnosis and endodontic treatment planning for cases with dystrophic calcifications and other types of canal obliteration [9, 11]. Recently, Gabardo et al. found an association between pulp stones and kidney stones [12]. It is important to emphasize that pulp stones are different from calcific metamorphosis (or PCO) but may demand the use of guided endodontics to gain access to the root canals, especially for adhered pulp stones not easily removed by ultrasonic instruments [13]. In PCO, the process of calcification can start with a reduction of the tubular lumen diameter and evolve to a partial or complete obliteration of the pulp chamber and root canals [2]. Root canal treatment in cases with PCO can be considered the main indication for guided endodontic access until then.

In this context of pulp canal obliteration and guided endodontics evolution, we must highlight the technological advances of the Cone Bean Computed Tomography (CBCT), intra-oral scanner, CAD-CAM software, and the stereolithography 3D printers that enabled the ideation of guided endodontics. The fidelity of CBCT images led to more accurate diagnostic in Endodontics [14]. The own Digital Imaging and Communication in Medicine (DICOM) standard network protocol must be considered a breakthrough technology. DICOM defines the operation of service classes beyond the simple transfer of data and creates a mechanism for uniquely identifying information objects as they are acted on across the network [15]. Among the technological evolution, the STL (Standard Triangle Language or Standard Tessellation Language) allowed the transformation of raw data into a representation of bone and dental structures in the 3D geometry of microscopic triangled surfaces [16]. The DICOM segmentation and STL processing at the CAD software enable better visualization of the root canal system, soft tissues, obliteration extension, and localization of the remaining canal' lumens. Therefore, the CBCT and the CAD-CAM automation in Endodontics promoted accuracy in diagnosing pulp calcifications and caused a paradigm shift of what endodontists recognize as a challenge to the specialty: risky and time-consuming access to obliterated pulp canals, for a quick treatment with less likelihood of errors.

Since 2016, guided endodontics access in clinical cases of PCO has gained greater attention [17]. However, the literature is still scarce, especially about guided endodontic access in molars. The challenge to treat obliterated root canals is even higher in posterior teeth [2]. Endodontic access in obliterated root canals of molars represents an increased risk of accidents such as perforation of the pulp chamber floor or the walls of the root canals [18]. The guided endodontics in molars demands more complex planning at the CAD phase because of the diverse anatomy. In addition, the limited field of view and demanding access to the molar region are considerable complications [19]. Despite the obliteration of the pulp canal, the individual of this case report had additional complications: the lower molar with the destruction of the crown and loss of reference as cuspids, the inclination of the tooth in the dental arch, and limited aperture of the mouth at the molar region. The primary aims of this case report were to describe the CAD-CAM workflow to create personalized templates with innovative design and the guided endodontics' clinical procedures to treat the obliterated mesiobuccal (MB) and mesiolingual (ML) root canals of a mandibular molar. Secondary aims were to compare the open-template design to the compact design and report the success assessment after one year of follow-up. This case report followed the CARE guidelines [20].

## Case presentation

**Fig. 1:**
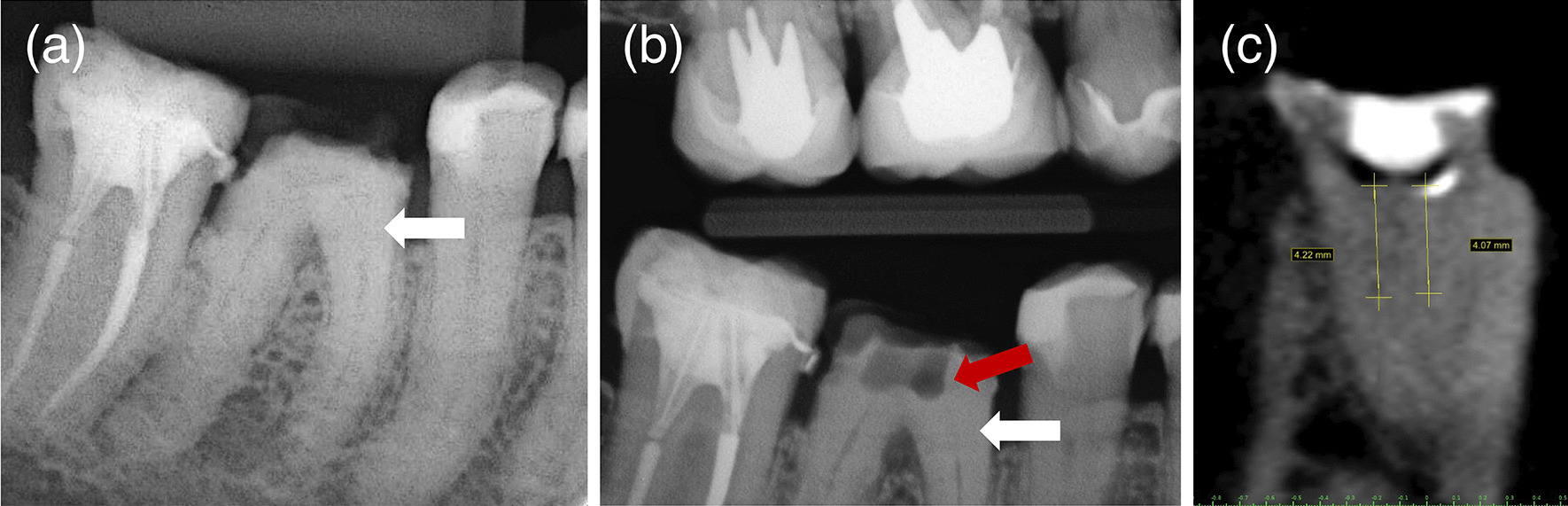
Diagnostic periapical XR image was suggestive of obliteration of the pulp canals (white arrow) (**a**). Bitewing XR image showing deviation in the mesial root (red arrow) towards the furcation area and the extension of the root canal obliteration (white arrow) (**b**). Cone Beam Computer Tomography (CBCT) image with estimated measurements of the obliteration’s length from the pulp chamber floor assuming the length of the 1.3 mm drill as the measurement parameter (**c**)

**Fig. 2:**
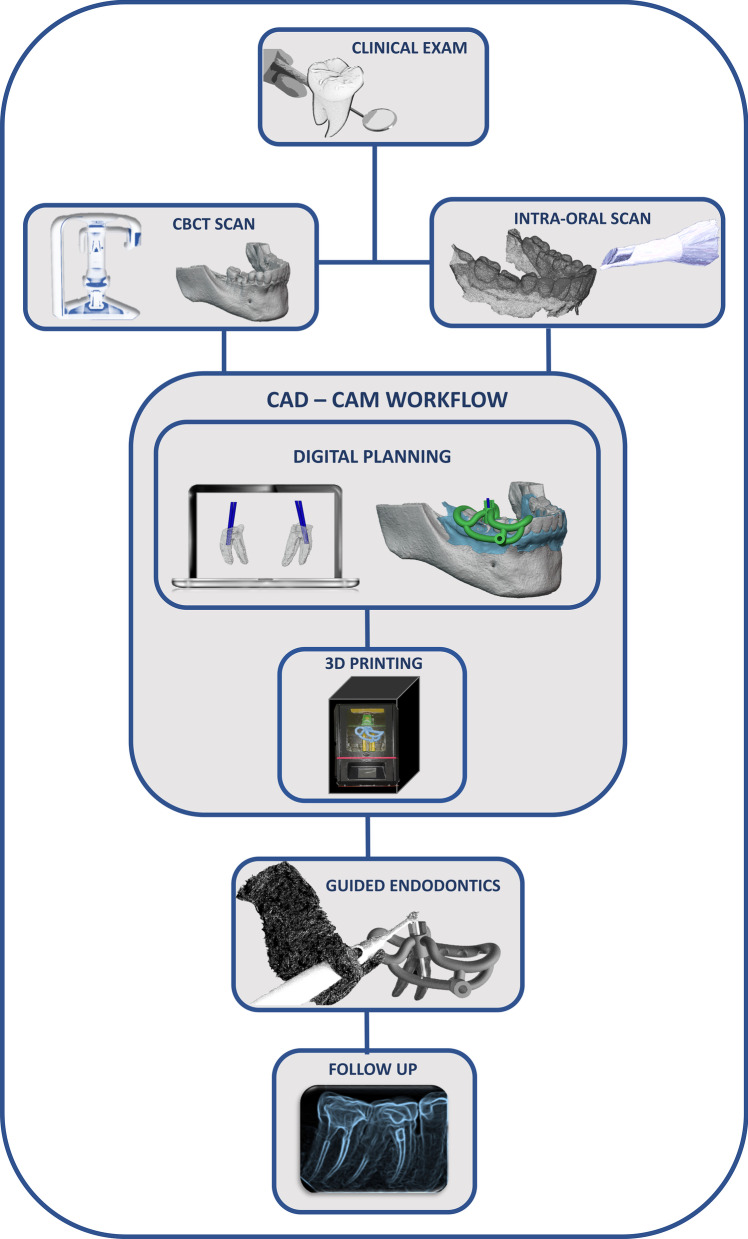
The flowchart illustrates the guided endodontic treatment of the study, from the clinical exam to the CAD-CAM workflow (Computer-Aided Design and Computer-Aided Manufacturing), root canal treatment, and one-year follow-up. This image was developed by the own authors

A 58-year-old female patient, leucoderma, attended the dental office in May 2020 for the endodontic treatment of the first right mandibular molar. The necessity of intraradicular posts and prosthetic restoration were the indications for endodontic treatment. The patient did not report any systemic disease (ASA I) or continuous medication usage. This study has been conducted in full accordance with the ethical principles of the World Medical Association Declaration of Helsinki (version 2008). All experiments, clinical procedures, exams, and photographs were undertaken with the understanding and written consent of the patient, according to the principles mentioned above. This case report was independently reviewed and approved by the ethical board committee of the Faculty of Health Sciences of the University of Brasilia (protocol No.4.767.576, Plataforma Brasil). Clinically, the patient was asymptomatic, with a negative response to thermal and percussion tests in the right first mandibular molar. The diagnostic intraoral radiography suggested obliteration of the root canals in the mesial and distal roots (Fig. 1). The patient did not report any trauma in the tooth indicated for the endodontic treatment. The dental record comprised a history of caries, prosthetic crowns, and root canal treatment in other teeth. There was no apical lesion visible at the periapical radiography. An attempt was made to access the root canals by conventional means. However, even with the aid of an operating microscope (Alliance microscopy—São Carlos, SP, Brazil), a deviation in the mesial root towards the furcation area occurred during the attempt to access the MB (Fig. 1). The tooth was inclined without crown references, and the patient buccal aperture at the molar region was limited. Therefore, the decision favored digital planning and the guided endodontic treatment aiming to avoid the risk of root canal perforation (Fig. 2).

The buccal, lingual, and inferior alveolar nerves were anesthetized first to treat the distal root canal. Briefly, the distal root canal was located under microscopy, using an E5 ultrasonic insert for the root canal debridement (Helse Ultrasonic, Santa Rosa de Viterbo, SP, Brazil) and under rubber dam isolation. The access to the distal root canal was successful, but it took approximately 30 min to accomplish. The diagnosis of pulp vitality was confirmed. After the working length establishment, pulp removal, and the distal root canal glide path, the endodontic treatment was completed using the medium file and the respective gutta-percha point of the WaveOne Gold system (Dentsply Maillefer, Balaigues, Switzerland). The endodontic sealer was the AH Plus (Dentsply Maillefer). The pulp chamber was filled with glass ionomer cement (Vitremer, 3 M Corporate Headquarters, St. Paul, MN, USA), and the crown was temporarily restored. After that, the patient was referred to a diagnostic imaging center for a CBCT acquisition. The necessity of the CBCT to establish the accurate diagnoses of the root canal obliterations and the digital planning of an endodontic template was informed to the patient. The risks related to the CBCT exam were also informed, and the patient signed the consent form.

The CBCT Scan of the mandible was performed in the i-CAT equipment (Imaging Sciences International, Xoran Technologies Inc., Hatfield, USA) with the following configurations: 160 × 160 × 54 mm FOV (Field of View), 0.2 × 0.2 × 0.2 mm Voxel, 120 kV, and 5 mA. The CBCT exam followed the guidelines of the European Society of Endodontology [21]. An area of tomography comprising the neighboring teeth from the same arch as the target tooth is mandatory for precise digital planning of the template. It is essential to emphasize that a lip retractor must be used during the CBCT scan to better segmentation of the DICOM and overlap with the scan data. The CBCT images confirmed the obliterations inside the mesiobuccal canal (MB) and the mesiolingual canal (ML) (Fig. 1). The estimated depths of the calcifications would pose a mighty risk of root canal perforation if the accesses were performed free-handed. The CBCT images confirmed the diagnoses of dystrophic calcification, and the guided endodontics as the best choice to access the root canals.

We also performed an intraoral scanning of the entire mandibular arch with the TRIOS 3 Basic equipment (3Shape A/S, Copenhagen, Denmark). Data on the teeth structure and oral mucosa are fundamental for designing the endodontic template (guide). The DICOM (Digital Imaging and Communication in Medicine) and STL (Standard Triangle Language) data from the CBCT and intraoral scan, respectively, were processed in a free digital planning software BlueSkyPlan (BlueSkyBio, Illinois, Us). The overlap between these two files was performed with maximum precision, a fundamental condition for the CAD-CAM workflow. The CAD phase comprised two steps: planning the drill's glide path based on the DICOM image data and the design of the template body using the STL file with the match of the intra-oral scanned image (Fig. 2).

**Fig. 3:**
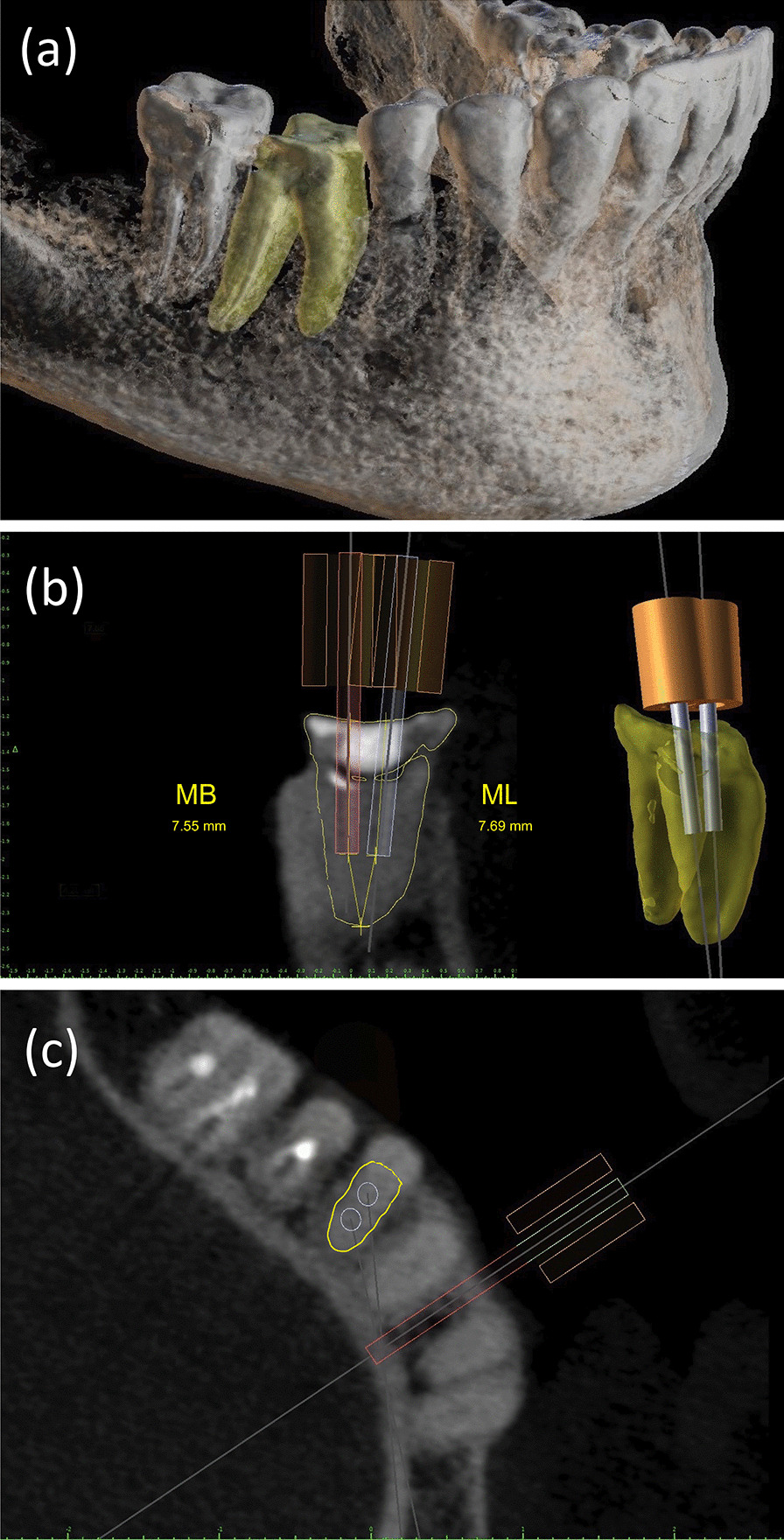
The DICOM data is segmented in an STL file for the 3D reconstruction of the mandible; the mandibular first molar with dystrophic calcification is in yellow (**a**). The CBCT image shows a buccolingual view of the obliterated mesial root with the virtual drills and respective virtual sleeves positioned in the access pathway (**b**). MB is the mesiobuccal, and ML is the mesiolingual root canal, respectively. The image also shows an estimated length of the obliterations from the flor of the pulp chamber to the remaining lumens (**b**). The axial view shows the virtual sleeve and drill for the fixation pin (**c**)

**Fig. 4:**
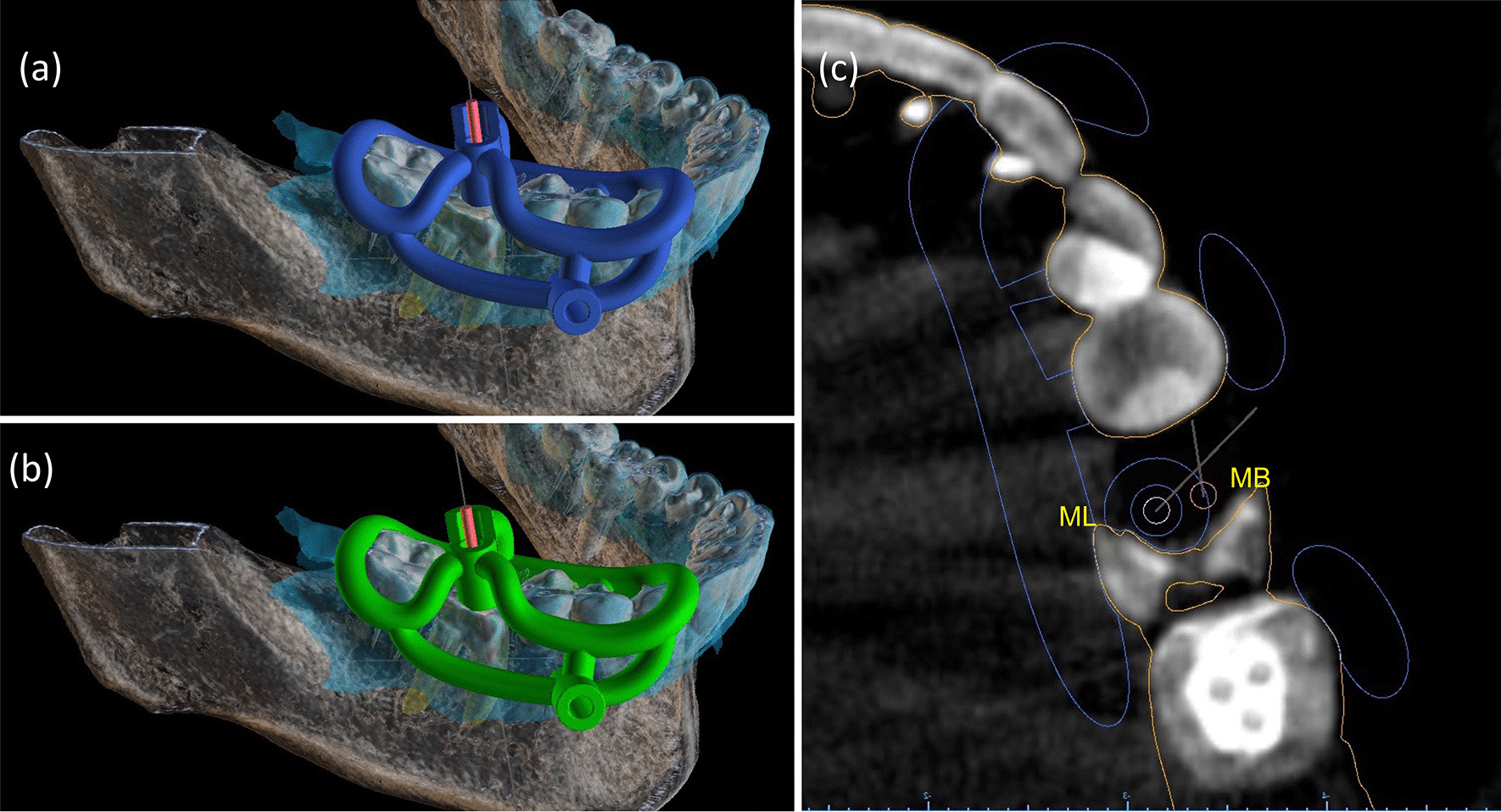
Both MB and ML templates followed an open design concept. The light-blue image represents the scanned area. The virtual ML template with drill and sleeve (**a**). The virtual MB template with the drill and respective sleeve (**b**). The resin base of metal sleeves was designed as a half-cylinder to facilitate the insertion of the drill. The top worked as a reference for the drilling length (**a** and **b**). The sleeve for the fixation pin had the same position for both templates, avoiding unnecessary bone drilling. CBCT image in the axial plane with virtual drills and sleeve of the ML template (**c**)

**Fig. 5:**
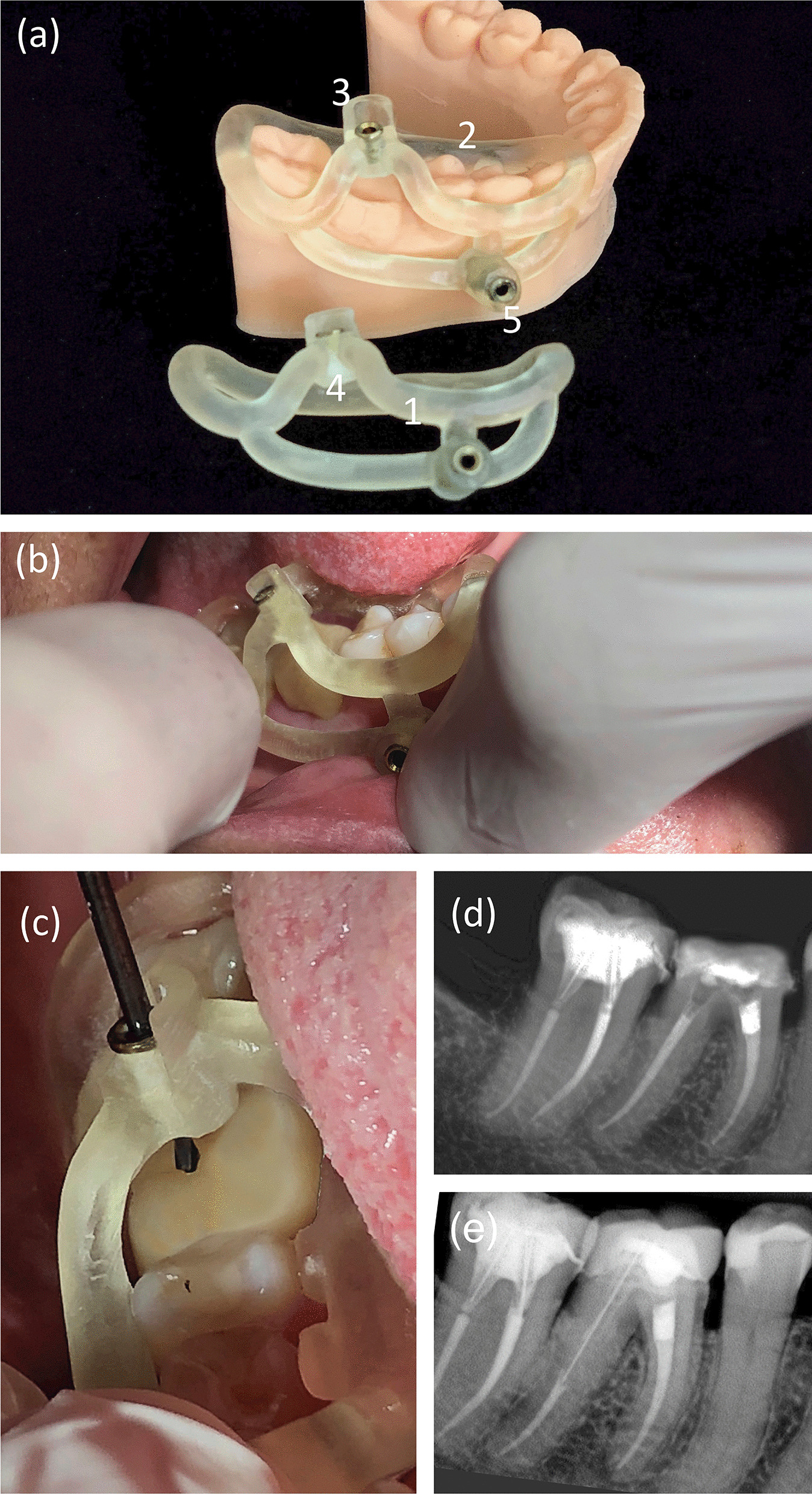
The two 3D-printed MB and ML templates and optional training model of the patient’s low dental arch (**a**). 1, Handles of the open template; 2, lock-arms that fit the target tooth and the neighboring teeth to increase the stability of the guide; 3 the top of resin cylindric bases of the sleeves as a reference for the working length of the drill; 4, the sleeves’ resin cylindric bases; 5, the sleeve for the fixation pin. The template stability must be previously evaluated (**b**). The drill was positioned inside the metal sleeve following the access direction as planned in the CAD phase (**c**). Final XR image after the guided access and endodontic treatment (**d**). The XR image of one-year follow-up (**e**)

First, we performed the DICOM segmentation using the BlueSkyplan software and created the 3D STL file (Fig. 3). Thereafter, the internal anatomy of the root canal system was observed. The mesial root of the right mandibular first molar showed remaining lumens at the apical third (Fig. 3). Virtual cylinders representing the 1.3 mm drill, 20 mm length (Straumann, Basel, Switzerland), were positioned across the obliterated areas to reach the beginning of the remaining lumens of MB and ML (Fig. 3). The working lengths of the drill inside the root canals were estimated at 7.55 mm (MB) and 7.67 mm (ML), considering the drill length as a parameter. The virtual drill orientation represents the exact path that the drill will take during the clinical procedure. The sleeve fixation pin was planned in the same position for both templates (Fig. 3); this strategy provided stability to the template avoiding extra bone drilling. Once the virtual drill and sleeves were designed and positioned, the STL file was exported from the BlueSkyplan to a second software (Blender for Dental, Gold Coast, Australia). Two templates were designed to guarantee independent access and the cleaning and shaping of each mesial root canal, preserving dental structure (Fig. 4). Both resin bases of the guidance sleeves were designed with the shape of half-cylinders, aiming to facilitate the positioning of the drill into the sleeve because of the limited mouth opening in the molar region (Fig. 4). We planned the tops of the sleeves resin base from both templates to limit the working length of the drill and promote security to the operator, a strategy to prevent inadvertent drilling in the danger zone of the mandibular molar at the root curvature. It is indispensable to mention that a minimum clearance of 0.05 mm between the virtual templates and dental structures was respected to compensate for software shape error and avoid future excessive pressure of the 3D printed templates over the dental structures. After completing the virtual design of the templates, the CAM phase started with the STL files export to the printer software MiiUtility V 6.3.0 (Young Optics Inc., Hsinchu Science Park, Hsinchu, Taiwan). The templates were printed with the Smart Print Bio Bite Splint resin (MMTech Projetos Tecnológicos Importação e Exportacao Ltda, SP, Brazil), using a 3D printer MiiCraft Ultra 125 (Young Optics Inc.) (Fig. 5). A space between the tooth surface and the bottom of the sleeve was intentionally left to allow the escape of dentin debris. To enhance the stability of the endodontic template, we added small arms that act as stability clamps on the neighboring teeth.

The templates were previously tested over the patient's teeth before the guided endodontics procedure (Fig. 5). The patient was anesthetized by blocking the inferior alveolar, lingual, and buccal nerves. Subsequently, the endodontic guide of the ML canal was positioned and fixed with the fixation pin. The access to the canal was performed with the 1.3 mm drill (Straumann) coupled to the XSmart Plus engine (Dentsply Maillefer) calibrated at 1200 RPM and 4 N (Fig. 5). The 1.3 drill was placed inside the metal sleeve, the XSmart Plus was triggered, and with “pecking” movements, the drill gained access, overcoming the calcification. The drilling procedure was constant until the entire working length as planned at the CAD phase and followed by irrigation with saline solution to avoid the heating of the drill. The first endodontic template was removed and replaced by the MB canal one. After accessing the two mesial canals, the template was removed, and the tooth was isolated with a rubber dam and dental clamp 206. The glide paths of the MB and ML were performed with a 10 Kerr file (Dentsply Maillefer, Balaigues, Switzerland). The working length of 20 mm was established with a NovApex foraminal locator (Romidan, Kiriaty Ono, Israel) for both MB and ML. The root canals were instrumented with the Primary file of the WaveOne Gold reciprocating system (Dentsply Maillefer) under 2.5% sodium hypochlorite irrigation. The smear layer was removed by final irrigation with 17% EDTA and activation with the Easy Clean device (Easy, Belo Horizonte, Brazil) for 30 s.

Finally, the root canals were irrigated with saline solution and dried with sterile absorbent paper cones (Dentsply Maillefer). The MB and ML root canals fillings were performed with primary cones (WaveOne) using AH Plus as an endodontic sealer (all from Dentsply Maillefer). The tooth was temporarily restored, radiographed and the patient was referred for prosthetic rehabilitation. The final periapical radiography showed respect to the mesial root canal anatomy and a root canal filling following the endodontic standards for a successful treatment (Fig. 5). By the end of the guided endodontic treatment, the patient was questioned about adverse effects and satisfaction with the procedure: level of pain on a scale of 0–10, heating of the tooth region, or any discomfort, how confident and would recommend this type of treatment. The patient reported no adverse effects and expressed satisfaction with the treatment, and the answers were registered on the patient dental record. After one year of follow-up, the tooth was asymptomatic, with a prosthetic crown adapted, negative to percussion tests, and the radiographic image showed the integrity of the adjacent tissues (Fig. 5).

## Discussion and conclusion

This clinical report is a successful case of guided endodontic treatment in a mandibular molar with dystrophic calcification. Two templates were customized to position the drill in a region of limited mouth opening to gain access to the MB and ML root canals. Moreover, instead of a close template, we used an innovative open design concept for both templates that facilitated direct irrigation and visualization of the working area. Endodontic treatments in molars with obliterated root canals are complex because of the tooth anatomy and posterior location. The mesial roots of mandibular molars are usually narrowed, curved, and have two remarkably close canals that make endodontic access difficult in this group of teeth, especially when obliterated. The dystrophic calcification of the mesial root canals of this case report extended to the beginning of the medium third. The attempt of conventional endodontic access posed a mighty risk of deviation and perforation in the furcation area due to the inclination of the tooth and narrowing of the root.

Until 2017, researchers and clinicians believed that guided endodontics might not be possible in molars because of the limited space for the template and the drill in the posterior region [22, 23]. Our case report demonstrated strategies adopted in the CAD-CAM workflow that allowed us to overcome those difficulties. Recently, authors reported the switch for guided endodontics after root perforations during the attempt to access the mesial and distal canals of a molar with type 1 dentin dysplasia (DD1) in a 12-year-old patient [5]. Different from the molar of this study, the DD1 tooth presented straight shortened roots and aspects of a single canal in the mesial root. Our case report proves that the guided endodontics technique can also be used in obliterated complex mesial root canals of mandibular molars, providing precision and safety. In 2016, van der Meer et al. proposed a digital planning protocol for endodontic access in anterior teeth inspired by implantology protocols [24]. Closed templates probably dominated the guided endodontics technique due to technology transfer from the dental implantology software [5, 25, 26]. The closed or compact templates cover the teeth crowns, limiting the field of view and decreasing irrigation capacity and debris removal during drilling [27]. Misir et al. showed that closed templates generated significantly higher temperatures than classical preparation techniques during bone drilling. The authors speculated that the difference in temperature increase between the techniques was because the closed design did not allow the irrigation solution to reach the preparation sites [27]. The generation of increased temperatures on the root surface during drilling represents a potential insult to the periodontal ligament and the adjacent bone [28].

Furthermore, the closed template demands a large contact area with the adjacent teeth and consequently more significant internal surface relief. Such characteristics can contribute to the impairment of field visualization and compromise the template stabilization, respectively [29]. Verification windows are essential to visualize the template's correct adaptation on the teeth. We designed an innovative concept of an open template with rod handles that contoured and self-locked the teeth crowns to overcome the disadvantages of traditional templates. The design allowed direct view and irrigation of both drill and new entrances of the root canals, favoring the instrument cooling and smear-layer removal during the guided access. We also created lock-arms that fitted the target tooth and neighboring teeth. Both rod handles and lock-arms were designed with a minimum clearance of 0.05 mm to ensure the template "self-locking" and increase its stability. The strategy was suitable to avoid balancing movements during the drilling. The self-locking occurred between the rod handles and the equator of the tooth crown. Other authors recently proposed a different open and sleeveless design for an endodontic template to access an upper pre-molar [30]. Their template involved teeth of the whole superior arch. According to the authors, their design could be uncomfortable for some patients and difficult to use in the molar region. The endodontic template used in this case reports successfully led to access to the mandibular molar's MB and ML root canals and involved only four neighboring teeth. The design also minimized the interference of the patient's tongue and lips. Moreover, even with few teeth involved and reduced clearance, our design provided precision of the insertion axis, adaptation, and high stability. It is essential to highlight that the merging of CBCT and intraoral scan images of the patient at the CAD phase increased the precision of the endodontic template as already described in an ex-vivo study [31].

When planning the endodontic templates, the association of CBCT and intraoral scanner images enables a more predictable and safer intervention [32]. Compared with the sleeveless proposal, we favor using metal sleeves because it will adequately guide the drilling pathway without the risk of resin damaged by over-heating or undesirable resin drilling, as could happen with simple orifices to introduce the drill. The perfect adaptation between the drill and sleeve of the 3D-printed template minimizes the possible deviation of the instrument. Such deviations are mainly related to the depth and angulation of the perforation [31]. Due to the limited mouth opening in the posterior region, we designed the resin base of the drill sleeve with the shape of a half-cylinder at the upper part to facilitate the insertion of the drill. The templates of this case report were highly stable, and the fixation pin could be despised. However, the stabilization pin prevented manual stabilization and possible interferences of the patient's lips or tongue. In the posterior region, the risk of displacement of the template due to movements of the buccal mucosa, oral muscles, and tongue is higher, and fixation of the template should always be recommended.

The guided endodontics technique applied in this case report has its limitations, for example, the need for prior training. Like all techniques, the guided endodontics demands a learning curve, even if it is fast and straightforward. Another limitation of the technique is the impossibility of urgency procedures because it requires CBCT and intra-oral scanning in advance. The patient's exposure to radiation during the CBCT scan can be considered a concerning point as well. According to the SEDENTEXCT guidelines [33], the CBCT is not indicated as a standard method to scan the anatomy of the root canal. Therefore, the CBCT for guided endodontics must have an accurate indication, such as multi-rooted teeth with canal obliterations that conventional intraoral radiographs provide insufficient information on the anatomy to establish an adequate treatment plan. For instance, the limited volume and high-resolution CBCT for guided endodontics may aid in identifying relationships with critical anatomical structures and management of pathogenic anatomies like dystrophic calcification [33]. The guided endodontic treatment demands unique equipment and devices. However, specialized digital planning centers can take over the CAD-CAM workflow and bypass the need for endodontists to acquire software, computers, and 3D printers. Most software, drills, and sleeves used for guided endodontics originated from implantology [34]. The 1.3 mm drill was formally developed for dental implants but was successful in other case reports of guided endodontics [16]. Larger diameter drills suffer a lower degree of flexion; however, parsimony is required in this choice to prevent an unnecessary cut of dentin that may cause tooth fragility. New instruments specific for guided endodontics are emerging, as drills with smaller diameters [35]. However, some features are necessary as bending resistance and specific sleeves with minimum clearance to restrain the drill deviations. In addition, the active part and design of the drill are still a subject little discussed in the literature. Some instruments were designed with long active parts, impairing their usage in specific teeth like molars.

When planning the guided endodontic access, it is also essential to consider the type of tissue that will be drilled. Enamel, dentin, bone, and dental materials may require specific drills for each case. In this clinical case, the two independent templates allowed safe access to the two mesial canals of the mandibular molar, reducing the risk of possible accidents such as perforations and even over-drilling of the root canal walls. The working time was considerably reduced from hours and possibly multiple sessions to 10 min to access the mesial root canals. After one year of follow-up, the endodontic treatment was successful.

The patient was surprised with the speed of access by guided endodontics compared with the conventional technique in the distal canal. There was no complaint of discomfort due to the fixation pin. On the contrary, the patient manifested to be comfortable and satisfied with the innovative approach.

Guided endodontics was initially proposed for cases of anterior teeth with dental anomalies or calcific metamorphosis. Our case report demonstrated that guided endodontics was a successful technique for accessing obliterated root canals, even in more complex cases such as the mesial canals of mandibular molars. The digital plan for treating the mandibular molar with dystrophic calcification reinforces guided endodontics as personalized treatment. The knowledge of anatomy and creativity in templates design led to automation, safety, and precision to access the root canals in a region of limited mouth opening. The "digital future" holds an increase in the success of endodontic treatments.

## Data Availability

The datasets used and/or analysed during the current case report are available from the corresponding author on reasonable request.

## Abbreviations

CAD–CAM: Computer-Aided-Design and Computer-Aided-Manufacturing
DICOM: Digital Imaging and Communications in Medicine
STL: Standard Triangle Language or Standard Tessellation Language
3D-printed: Three-dimensional printed
PCO: Pulp canal obliterationtion
CBCT: Cone-beam computed tomography
ASA I: American Society of Anesthesiologists risk I (healthy person)
MB: Mesiobuccal root canal
ML: Mesiolingual root canal
DD1: Dentin dysplasia type 1

## References

[1] Kfir A, Telishevsky-Strauss Y, Leitner A, Metzger Z (2013). The diagnosis and conservative treatment of a complex type 3 dens invaginatus using cone beam computed tomography (CBCT) and 3D plastic models. Int Endod J.

[2] McCabe PS, Dummer PM (2012). Pulp canal obliteration: an endodontic diagnosis and treatment challenge. Int Endod J.

[3] Clark D, Levin L (2019). Prognosis and complications of mature teeth after lateral luxation: a systematic review. J Am Dent Assoc.

[4] Kapferer-Seebacher I, Schnabl D, Zschocke J, Pope FM (2020). Dental manifestations of Ehlers–Danlos syndromes: a systematic review. Acta Derm Venereol..

[5] Krug R, Volland J, Reich S, Soliman S, Connert T, Krastl G (2020). Guided endodontic treatment of multiple teeth with dentin dysplasia: a case report. Head Face Med.

[6] Bauss O, Neter D, Rahman A (2008). Prevalence of pulp calcifications in patients with Marfan syndrome. Oral Surg Oral Med Oral Pathol Oral Radiol Endod.

[7] Goga R, Chandler NP, Oginni AO (2008). Pulp stones: a review. Int Endod J.

[8] Velmurugan N, Kasabwala KA, Saumya-Rajesh P, Ashritha M (2020). Pulp canal obliteration: a review. J Oper Dent Endod.

[9] Maeda H (2020). Aging and senescence of dental pulp and hard tissues of the tooth. Front Cell Dev Biol..

[10] Estrela C, Decurcio DDA, Rossi-Fedele G, Silva JA, Guedes OA, Borges ÁH (2018). Root perforations: a review of diagnosis, prognosis and materials. Braz Oral Res.

[11] Mahmud KH, Iqbal MA, Talukder FH (2020). Management of Calcific metamorphosis by conventional root canal treatment: a case report. Update Dent Coll J..

[12] Gabardo MCL, Wambier LM, Rocha JS, Küchler EC, de Lara RM, Leonardi DP, Sousa-Neto MD, Baratto-Filho F, Michel-Crosato E (2019). Association between pulp stones and kidney stones: a systematic review and meta-analysis. J Endod.

[13] Jannati R, Afshari M, Moosazadeh M, Allahgholipour SZ, Eidy M, Hajihoseini M (2019). Prevalence of pulp stones: a systematic review and meta-analysis. J Evid Based Med.

[14] McClammy TV (2014). Endodontic applications of cone beam computed tomography. Dent Clin North Am.

[15] Kahn CE, Langlotz CP, Channin DS, Rubin DL (2011). Informatics in radiology: an information model of the DICOM standard. Radiographics.

[16] Kamio T, Suzuki M, Asaumi R, Kawai T (2020). DICOM segmentation and STL creation for 3D printing: a process and software package comparison for osseous anatomy. BMC 3D Print Med.

[17] Moreno-Rabié C, Torres A, Lambrechts P, Jacobs R (2020). Clinical applications, accuracy and limitations of guided endodontics: a systematic review. Int Endod J.

[18] Vehkalahti MM, Swanljung O (2020). Accidental perforations during root canal treatment: an 8-year nationwide perspective on healthcare malpractice claims. Clin Oral Investig.

[19] Maia LM, de Carvalho MV, da Silva NRFA, Brito Júnior M, da Silveira RR, Moreira Júnior G, Ribeiro Sobrinho AP (2019). Case reports in maxillary posterior teeth by guided endodontic access. J Endod.

[20] Riley DS, Barber MS, Kienle GS, Aronson JK, von Schoen-Angerer T, Tugwell P, Kiene H, Helfand M, Altman DG, Sox H, Werthmann PG, Moher D, Rison RA, Shamseer L, Koch CA, Sun GH, Hanaway P, Sudak NL, Kaszkin-Bettag M, Carpenter JE, Gagnier JJ (2017). CARE guidelines for case reports: explanation and elaboration document. J Clin Epidemiol.

[21] Patel S, Brown J, Semper M, Abella F, Mannocci F (2019). European Society of Endodontology position statement: use of cone beam computed tomography in Endodontics: European Society of Endodontology (ESE) developed by. Int Endod J.

[22] Fonseca Tavares WL, Diniz Viana AC, de Carvalho MV, Feitosa Henriques LC, Ribeiro Sobrinho AP (2018). Guided endodontic access of calcified anterior teeth. J Endod.

[23] Connert T, Zehnder MS, Weiger R, Kühl S, Krastl G (2017). Microguided endodontics: accuracy of a miniaturized technique for apically extended access cavity preparation in anterior teeth. J Endod.

[24] van der Meer WJ, Vissink A, Ng YL, Gulabivala K (2016). 3D Computer aided treatment planning in endodontics. J Dent.

[25] Zehnder MS, Connert T, Weiger R, Krastl G, Kühl S (2016). Guided endodontics: accuracy of a novel method for guided access cavity preparation and root canal location. Int Endod J.

[26] Lara-Mendes STO, Barbosa CFM, Santa-Rosa CC, Machado VC (2018). Guided endodontic access in maxillary molars using cone-beam computed tomography and computer-aided design/computer-aided manufacturing system: a case report. J Endod.

[27] Misir AF, Sumer M, Yenisey M, Ergioglu E (2009). Effect of surgical drill guide on heat generated from implant drilling. J Oral Maxillofac Surg.

[28] Hussey DL, Biagioni PA, McCullagh JJ, Lamey PJ (1997). Thermographic assessment of heat generated on the root surface during post space preparation. Int Endod J.

[29] Perez C, Finelle G, Couvrechel C (2020). Optimisation of a guided endodontics protocol for removal of fibre-reinforced posts. Aust Endod J.

[30] Torres A, Lerut K, Lambrechts P, Jacobs R (2021). Guided endodontics: use of a sleeveless guide system on an upper premolar with pulp canal obliteration and apical periodontitis. J Endod.

[31] Buchgreitz J, Buchgreitz M, Mortensen D, Bjørndal L (2016). Guided access cavity preparation using cone-beam computed tomography and optical surface scans: an ex vivo study. Int Endod J.

[32] Anderson J, Wealleans J, Ray J (2018). Endodontic applications of 3D printing. Int Endod J.

[33] The SEDENTEXCT consortium. Radiation protection No 172. Cone beam CT for dental and maxillofacial radiology. Evidence based guidelines. https://www.sedentexct.eu/content/guidelines-cbct-dental-and-maxillofacial-radiology.htm. Accessed 01 January 2022

[34] Ahn SY, Kim NH, Kim S, Karabucak B, Kim E (2018). Computer-aided design/computer-aided manufacturing-guided endodontic surgery: guided osteotomy and apex localization in a mandibular molar with a thick buccal bone plate. J Endod.

[35] Loureiro MAZ, Silva JA, Chaves GS, Capeletti LR, Estrela C, Decurcio DA (2021). Guided endodontics: the impact of new technologies on complex case solution. Aust Endod J.

